# Mediating Role of Anxiety and Depression in the Relationship Between Posttraumatic Stress Disorder and Aggression in Motor Vehicle Accident: A Structural Equation Modelling Approach

**DOI:** 10.30476/beat.2020.85690

**Published:** 2020-10

**Authors:** Fatemeh Sadat Asgarian, Mahshid Namdari, Hamid Soori

**Affiliations:** 1 *Safety Promotion and Injury Prevention Research Center, Shahid Beheshti University of Medical Sciences, Tehran, Iran*; 2 *Department of Community Oral Health, Dental School, Shahid Beheshti University of Medical Sciences, Tehran, Iran.*

**Keywords:** Motor vehicle, Structural equation modelling, Posttraumatic stress disorder

## Abstract

**Objective:**

This study investigated the existence of anxiety and depression mediating effect on the relationship between PTSD and aggression in the hope of providing more comprehensive and effective trauma treatment in motor vehicle accident.

**Methods:**

The studied population of the study consisted of **motor vehicles **with posttraumatic stress disorder in Kashan. Three questionnaires of post-traumatic stress disorder, *Hospital Anxiety and Depression Scale (HADS) and Aggression Questionnaire (Buss & Perry, 1992)* were used for data collection. In order to analyze the mediating effects of anxiety and depression on the relationship between PTSD and aggression, structural equation modeling(SEM) was performed with** the maximum likelihood** **ratio as the method of estimation.**

**Results:**

Evaluation of the research hypothesis model using fitness indices showed that the hypothetical model fits with the measurement model. NFI=0.96, CFI =0.95, RMSEA=0.06 and the results showed that PTSD had indirect exacerbating effects on aggression. The results also confirmed the mediating role of anxiety and depression in the relationship between post-traumatic stress disorder and aggression in motor vehicle accident.

**Conclusion:**

The findings demonstrate that anxiety mediates the relationship between PTSD and aggression. Therefore, this finding can help to prioritize therapeutic goals and determine therapeutic focus for mental health professionals. It is possible to reduce one's aggression by focusing on his anxiety and increasing his/her ability to handle and manage it.

## Introduction

PTSD is one of the most common psychiatric disorders following accidents[1], with significant costs for the individual and society at large scale. Most of these disorders are caused by accidents[2]. In Iran, more than 51 percent of all traffic accidents that result in death or hospitalization occur for motorcyclists or motorcycle riders [3]. These victims are at risk for mental health problems such as PTSD, depression, and drug-related disorders[4]. Victims of motor vehicle accidents with PTSD experience more physical and psychological dysfunction [5].

**Those patients who have experienced traumatic events but do not have PTSD show lower rates of comorbidity with anxiety and affective disorders compared to PTSD patients with increased odds of mood (OR= 4.9) and anxiety (OR=4.3) disorders [** 6 **]** **.** **Strong correlations are suggested between PTSD symptoms and depression or anxiety in** **both acute stress disorder and PTSD[**7**]. People with PTSD often present with depressive and anxiety disorders[**8**]**. **Furthermore, the mediating effects of anxiety and depression in relationship between posttraumatic stress symptoms (PTSD) and aggression have not been documented in any study. Understanding the shared and unique effects of PTSD, anxiety, and depression on aggression will have important implications for treatment planning. The aim of the current study was to test this hypothesis that anxiety and depression have mediating effects on the relationship between PTSD and aggression among **motor vehicle accident**, using structural equation modeling. **

## Materials and Methods

**A cross-sectional study was conducted among motor vehicles drivers**. Structural equation modeling(SEM) is a powerful multivariate analysis method through which we can test hypotheses about causal relationships between "latent variables". The variables studied in this method are referred to as latent variables, observed variables, endogenous and exogenous variables. The latent variables are not directly observable and are measured by the observed variables. The exogenous variables are former variables and endogenous variables are mediating or dependent variables. A structural function model is examined with respect to the vectors drawn from exogenous variables to endogenous variables[9]. 

The statistical population of this study was injured motorcyclists referred to Shahid Beheshti hospitals in Kashan [10]. In the first stage, a list of people which have accident, was prepared and randomly selected and entered into the study provided the research conditions were met. In this study all traffic accidents and injuries that were referred to Kashan hospitals for medical services or were transferred to hospital by emergency department, other accidents that resulted in mortality at the scene of the accident or people who need to be hospitalized due to injuries were not included in the study. In this study, based on triage schedule, all patients with a history of accident between 6 weeks to 6 months ago which were assigned to triage levels of 3, 4, and 5 were randomly included.

 According to the structural equation guidelines, 300 samples were used in this study. 8 to 10 samples were used per parameter [11]. In order to observe ethical considerations in the research, the individual was assured of the confidentiality of information, confidentiality, freedom of association in the study, and their random selection.

We also asked the participants to read the questionnaires carefully and written informed consent to participate in the study was obtained. To collect demographic data a researcher-made questionnaire including: demographic information (age, education, driving history) and clinical anxiety and depression scale questionnaires, Buss and Perry aggression, and posttraumatic stress disorder was used. 

*Inclusion criteria*

Minimum middle school literacy level for ability to answer questions, experience of at least one year of motorcycling, no known mental illness based on the person's statement, desire and informed consent, age range of 18 to 65 years, ability to communicate and experience between 6 weeks to 6 months after accident.

*Exclusion criteria*

Past chronic illness, mental disorder (based on the person's statement) and mental retardation, level 1 and 2 triage patients, unwillingness to cooperate, patients with no registered address or outside of Kashan area were excluded from the study.

*Post-traumatic stress disorder*

A Self-Reporting Scale is used to evaluate post-traumatic stress disorder and screen patients as a diagnostic tool. The advantage of this listing is its short. The DSM-5 Diagnostic Criteria for the National Center for Post- Stress Disorder in united states is comprised of 20 questions, including 5questions for intrusive and unwanted symptoms, 2 questions for avoidance symptoms, 7 questions for negative changes in knowledge and creation, and 5 questions related to Signs of arousal and reactivity[12].

*Hospital Anxiety and Depression Clinical Scale (HADS)*

The Hospital Anxiety and Depression Clinical Scale (HADS) is a 14-item self-report tool designed to screen for the presence and severity of depression and anxiety symptoms in patients. The population of the study ranged from adolescents aged 16 years and more to elderly. The instrument has a seven-item depression subscale and a seven-item anxiety subscale. The depression subscale of the HADS questionnaire focuses on assessing the lack of happiness. Thus, the HADS questionnaire provides a useful and concise screening tool for symptoms of depression and anxiety in patients with physical problems. Each test component is scored on a scale of zero to three (0-3). Therefore, the scores on the subscale of depression and anxiety HADS questionnaire ranged from zero to 21. For both subscales, scores in the range of zero to seven are considered normal, eight to 10 mild, 11 to 14 moderate, and 15 to 21 severe[13,14]. Correlation coefficient of clinical quantitative evaluation of this questionnaire in Iran was calculated with Beck Depression Inventory (BDI) (r = 0.70, *p*<0.001) and Beck Anxiety Inventory (BAI) ((r = 0.72, *p*<0.001)). Also for Cronbach's alpha calculation, seven-item subscale of depression scale (Alpha = 0.70) and seven-item subscale of anxiety scale (Alpha = 0.85) were obtained.

*Buss and Perry Aggression Questionnaire*

This questionnaire assesses four behavioral factors: physical aggression (nine questions), verbal aggression (five questions), anger (seven questions), and malice (eight questions). These factors are categorized into three motor or instrumental components (physical and verbal aggression) emotional (anger) and cognitive (malice). The validity of the original form of this questionnaire was calculated as 0.80, 0.76 and 0.72 for the factors of physical, verbal aggression, anger and malice, respectively. This questionnaire was validated in Iran by Samani [15].

*Statistical analysis*

The data were analyzed using the Stata version 14.0. A structural equation modeling (SEM), a type of multivariate analysis, was applied in order to confirm the theoretically built model which includes the domains of working conditions, PTSD, anxiety and depression, and consequently, aggression. First, the model was designed and fitted with a well-defined research question. Then, the estimation and their significant levels for each parameter were obtained. Afterward, model diagnostics including measures of model fitness and modification indices were obtained. If indicated, correlations were added between error terms to improve the model. The chi-square statistic provides a test of the null hypothesis that the theoretical model fits the data. According to Jöreskog [16] suggestion the *p* value for this test of close fit should be more than 0.50. The criteria for model fit used were relative chi square statistic of less than or equal to 2.0, Goodness-of-Fit Index (GFI) statistic of equal to or greater than 0.95 [16], Comparative Fit Index (CFI) of equal to or greater than 0.90[17], and Root Mean Square Error of Approximation (RMSEA) of less than or equal to 0.8[18]. Finally, the estimates and significant levels of correlation and regression parameters from the fitted model were presented. Totally, direct and indirect effects of PTSD, anxiety and depression on aggression were calculated using the standardized regression weights of each pathway.

## Results

The study sample consisted of 300 motorcyclists with post-traumatic stress disorder with mean and standard deviation age 43 ±12 years and minimum age of 18 and maximum of 65 years.

The demographic characteristics and descriptive findings of the present study are presented in Table 1.

***Correlation coefficients***

Table 3 **shows the Pearson correlation coefficient matrix of the observed variables. PTSD is directly correlated with anxiety (** **r** **= **.539^**^, *p* **< 0.001), depression (**r=0.434^**^, *p* **< 0.001), and aggression **(r **= **.345^**^, *p ***< 0.001)**

***Final model***

**Fig.1:**
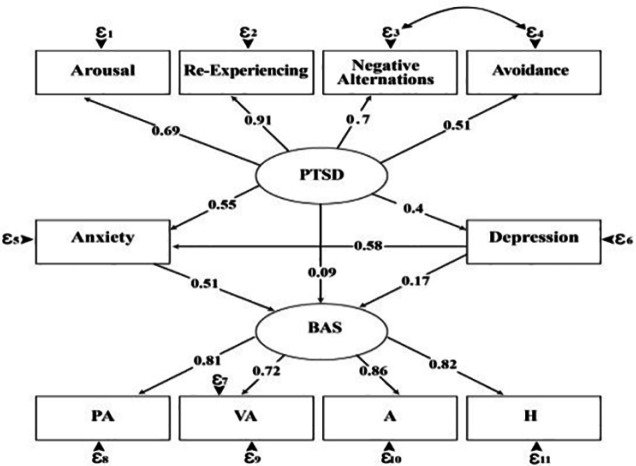
Final structural model: the mediating eﬀects of anxiety and depression between PTSD and aggression

As shown in Figure 1, the direct effect of post-traumatic stress disorder on aggression was not significant (P = 0.09). Post-traumatic stress disorder on depression had a direct effect of 0.4 (P<0.0001).

Anxiety had direct effect of 0.51 (*p*<0.0001) on aggression. Post-traumatic stress disorder does not have a significant effect on aggression (B=0.093, P=0.26). **Using the Sobel test, we discovered that the individual indirect effects of PTSD on aggression intrusiveness through anxiety, through depression and through depression to anxiety were significant** **(**β **= 0.14, **P **= 0.001; **β **= 0.098, **P **= 0.004; **β **= 0.089, **P **= 0.001respectively)** (Table4). 

**The** **measures of model fitness were as follows: chi square for** **Goodness-of-Fit test (χ2 = 22.80, **df **= 19, **p **= 0.246), relative chi square (1.200), CFI (0.95), TLT (0.92) and RMSEA (0.06)**

According to Table 5, the RMSEA value is 0.06, and since it is less than 0.1 indicates that the mean square of the model errors is appropriate and the model is acceptable. Also the GFI, CFI, and TLT indexes are greater than 0.9, indicating that the model for measuring the research variables is a good model (Table5).

**Table 1:** participant characteristics

Total		
Mean±SD	**N (%)**	**Variable**
		Sex
	300(100)	Male
43 ±12		Age
13±11		Driving history
		Marital status
	189(61.5)	Married
	9(2.9)	Divorced
	102(35.6)	Unmarried
		Educational level
	19(6.1)	Illiterate
	29(9.4%)	Primary
	131(42.4)	high school
	130(42.1)	University degree

**Table 2:** Means, standard deviations among variables modeled in structural equation modeling

	Mean	Std. Deviation
Anxiety	8.37	4.32
Depression	6.93	3.56
HADS	15.31	7.12
PTSD	46.60	12.90
BAS	82.00	19.38

**Table 3:** **Pearson correlation coefficient matrix of the measured variables**

BAS^c^	HADS	Depression	Anxiety	PTSD	
.345^a^ .000	**.544** ^a^ .000	**.434** ^a^ .000	**.539** ^a^ .000	**1**	**PTSD** ^b^ ** Pearson correlation** **Sig.(2-tailed)**

^a^Correlation is significant at the 0.01 level (2-tailed); ^b^PTSD = posttraumatic stress symptoms; ^c^BAS = Aggression Questionnaire (Buss & Perry).

PTSD = posttraumatic stress symptoms, BAS = Aggression Questionnaire (Buss & Perry).

**Table 4:** Individual indirect eﬀects of PTSD on BAS

Factors	Standardizes effect(SE)	P value
Anxiety	.14 (.031)	0.00
Depression	.098( .033)	0.004
Depression to Anxiety	.089(.019)	0.00
Total	0.707(0.132)	0.00

**Table 5:** Model fitness examination indexes

X^2^	P	TLT	CFI	PMR	RMSEA
109	0.00	.92	.95	.05	.06

## Discussion

The goal of medical science and health care workers is to promote health and alleviate pain and illness and prevent preventable disabilities and early death [2]. Today, a large part of problems in developing countries is the problem of traffic and driving situation which are relevant to physical and mental issues of the drivers. Such problems can be prevented provided that the mental and psychological conditions of the drivers are recognized and based on their needs provide them with accurate and comprehensive knowledge[2]. Providing a structural model to determine the mediating effects of anxiety and depression on the relationship between PTSD and aggression as mentioned in the present study is not the only and last model. Even if one model fits in with the available data, there are still many other models that can fit the data.

The results of this study show that the presented model is considered as a suitable model. Model variables such as anxiety, depression, post-traumatic stress disorder and aggression had a very good structural relationship with each. Structural equation modeling results showed that anxiety played a mediating role between post- traumatic stress disorder and aggression in motorcyclists. In general, the results of the structural equation modeling support the hypothesis of research based on the mediating role of anxiety on post-traumatic stress disorder and aggression. As can be seen in the figure, aggression has an indirect effect on the severity of post-traumatic stress disorder through anxiety. This study showed a positive and relatively strong correlation between the two with post-traumatic stress disorder. Many studies, including the present study, have reported a positive relationship between these two constructs. In a study in Vietnam, there was a statistically significant association between aggression and PTSD severity[19]. Basile KC also found that people with PTSD reported impulsive aggression[20, 21].

Various studies have shown the association of PTSD with many disorders, the most common of which is drug and alcohol abuse (51%-80%), mood disorders, especially depression (26%- 65%), anxiety disorders (30-60%)[22]. People affected by PTSD are more likely to be aggressive in relationships than those without PTSD, and there is evidence of increased hostility, involvement, and violence in families with PTSD parents[23].The findings of this study showed that depression has a mediating effect on the relationship between PTSD and aggression and the correlation between depression and post-traumatic stress disorder is 0.4.

In the Khodadadi study, patients with PTSD were significantly more depressed after the car accident than the non-affected group[24]. The results of the Blanchard et al study were in line with the present study so that among the survivors of the accident, those with post-traumatic stress disorder were significantly depressed, in fact, depression was observed in 53% of PTSD patients[25]. Kupchik *et al*. obtained similar results[26]. Roth *et al*. have suggested that post-traumatic stress disorder can have a direct and indirect relationship with depression[27].Irish et al., Schnyder et al. consider depression as a strong predictor of post-traumatic stress disorder one year after vehicle accidents[28,29].**However, the findings of both studies support the importance of examining numerous facets of psychopathology, including anxiety and depression, in order to understand the amount of aggression among trauma survivors. **Some** studies have proposed a dimensional structure for posttraumatic stress. They examined how that structural factor model relates to external measures** **of psychopathology, such as depression and anxiety[**30**, **31**].Our findings emphasize the importance of assessment and intervention for** **anxiety symptoms. In patients with PTSD an intervention** **effective for one symptom might also be beneficial in controlling other symptoms and could** **mitigate the inﬂuence of many symptoms on trauma survivors. Findings of the present study have important implications for clinical practice. It was found that anxiety** **had the largest direct and indirect effects on aggression in accident. Psychosocial interventions aimed at decreasing aggression and increasing participation in valued life activities are important strategies to help people with** **PTSD improve their subjective well-being.** Based on these explanations, we can illustrate in this article that anxiety is an initial excitement in response to an accident. People feel insecure in this situation; they experience the anxiety of dying and believe that they are vulnerable and that an accident and death can happen to them. This reaction is consistent because by accepting these facts one can protect oneself against future threats. But if one cannot tolerate these anxieties and facts and find fear and anxiety disturbing, annoying and unbearable, he will probably try to defend himself against this anxiety with a secondary excitement, which is anger and aggression. Anger and aggression give the individual a sense of domination over an environment that is at odds with anxiety and arises from an inability to conform to his or her expectations with reality. Therefore, one feels that if he is not angry, he has to endure the overwhelming anxiety associated with his own death. So he tries to avoid feelings, memories and thoughts associated with the incident by being angry.

**Finally, future research is needed for exploring specific mechanisms of these mediation effects and** **evaluate whether clinical interventions to treat anxiety or depression further minimize aggression and enhance subjective well-being in people who had accident. **This confirms their need for psychiatric services and affirms the need for services such as vocational and psychosocial rehabilitation. What is important is that health care workers working in hospitals need to examine these factors and symptoms in patients. Early diagnosis and subsequent referral for treatment of depression and anxiety can reduce the negative impact of these conditions on quality of life in long-term.

The present study, like any other study, has some limitations: a researcher-made test was used to measure the dependent variable. Although this test had high reliability and validity, its total validity was based on honesty and the accuracy of the subjects in answering the questions being asked. The main limitation of the present study was that this study was performed on samples of motorcyclists, so caution should be exercised in generalizing the results to other people. Other researchers by repeating this study in other areas can help generalize the results. Also, the expression of hypotheses as causal relations is purely on the basis of research background, and in cause and effect conclusion of the current research findings, caution should be exercised and the limitations of correlational research should be considered. Obviously, the study of the direct and indirect impact of the proposed research in this study through the implementation of experimental research and in the intervention designs can reveal the causal role of the variables proposed in this model.
